# Protocol for a parallel group, two-arm, superiority cluster randomised trial to evaluate a community-level complementary-food safety and hygiene and nutrition intervention in Mali: the MaaCiwara study (version 1.3; 10 November 2022)

**DOI:** 10.1186/s13063-022-06984-5

**Published:** 2023-01-30

**Authors:** Evans A. Asamane, Laura Quinn, Samuel I. Watson, Richard J. Lilford, Karla Hemming, Cheick Sidibe, Ryan T. Rego, Sami Bensassi, Youssouf Diarra, Samba Diop, Om Prasad Gautam, Mohammad Sirajul Islam, Louise Jackson, Kate Jolly, Kassoum Kayentao, Ousmane Koita, Buba Manjang, Susan Tebbs, Nicola Gale, Paula Griffiths, Sandy Cairncross, Ousmane Toure, Semira Manaseki-Holland

**Affiliations:** 1 Institute of Applied Health Research, University of Birmingham, Birmingham, UK; 2 University of Science, Techniques and Technology Bamako, Bamako, Mali; 3 Center for Global Health Equity, University of Michigan, Ann Arbor, USA; 4 Birmingham Business School, University of Birmingham, Birmingham, UK; 5 Water Aid, London, UK; 6 International Center for Diarrhoeal Disease Research, Dhaka, Bangladesh; 7 Ministry of Health, Banjul, Gambia; 8 School of Social Policy, Health Services Management Centre, University of Birmingham, Birmingham, UK; 9 School of Sport, Exercise and Health Sciences Loughborough University, London, UK; 10 Developmental Pathways for Health Research Unit (DPHRU), School of Clinical Medicine, University of the Witwatersr, Johannesburg, South Africa; 11 Faculty of Infectious and Tropical Diseases, London School of Hygiene and Tropical Medicine, London, UK

**Keywords:** Diarrhoeal disease, Hygiene, Cluster randomised controlled trial, Behaviour change

## Abstract

**Background:**

Diarrhoeal disease remains a significant cause of morbidity and mortality among the under-fives in many low- and middle-income countries. Changes to food safety practices and feeding methods around the weaning period, alongside improved nutrition, may significantly reduce the risk of disease and improve development for infants. We describe a protocol for a cluster randomised trial to evaluate the effectiveness of a multi-faceted community-based educational intervention that aims to improve food safety and hygiene behaviours and enhance child nutrition.

**Methods:**

We describe a mixed-methods, parallel group, two-arm, superiority cluster randomised controlled trial with baseline measures. One hundred twenty clusters comprising small urban and rural communities will be recruited in equal numbers and randomly allocated in a 1:1 ratio to either treatment or control arms. The community intervention will be focussed around an ideal mother concept involving all community members during campaign days with dramatic arts and pledging, and follow-up home visits. Participants will be mother–child dyads (27 per cluster period) with children aged 6 to 36 months. Data collection will comprise a day of observation and interviews with each participating mother–child pair and will take place at baseline and 4 and 15 months post-intervention. The primary analysis will estimate the effectiveness of the intervention on changes to complementary-food safety and preparation behaviours, food and water contamination, and diarrhoea. Secondary outcomes include maternal autonomy, enteric infection, nutrition, child anthropometry, and development scores. A additional structural equation analysis will be conducted to examine the causal relationships between the different outcomes. Qualitative and health economic analyses including process evaluation will be done.

**Conclusions:**

The trial will provide evidence on the effectiveness of community-based behavioural change interventions designed to reduce the burden of diarrhoeal disease in the under-fives and how effectiveness varies across different contexts.

**Trial registration:**

ISRCTN14390796. Registration date December 13, 2021

**Supplementary Information:**

The online version contains supplementary material available at 10.1186/s13063-022-06984-5.

## Administrative information

**Table:** 

**Title**	Protocol for a parallel group, two-arm, superiority cluster randomised trial to evaluate a community-level complementary-food safety and hygiene and nutrition intervention in Mali: The MaaCiwara study
**Trial registration**	ISRCTN14390796
**Protocol version**	1.1 07 Dec 2021 1.2 31 Mar 2022 – *Amended funding statement to include support from the National Institute for Health Research (NIHR) Applied Research Collaboration (ARC) West Midlands* 1.3 10 November 2022- Revised version following reviewers’ comments
**Funding**	Medical Research Council (MRC), UK Research and Innovation (UKRI) Global Challenges Research Fund (GCRF) MR/T030011/1. RJL and SMH are also supported by the National Institute for Health Research (NIHR) Applied Research Collaboration (ARC) West Midlands. The funder of this trial has no role in the trial design, conduct, collection of data, analysis or writing of trial papers. The views expressed are those of the author and not necessarily those of the NIHR or the Department of Health and Social Care
**Author details**	^1^ Institute of Applied Health Research, University of Birmingham, UK ^2^ University of Science, Techniques and Technology Bamako, Bamako, Mali ^3^ Center for Global Health Equity, University of Michigan ^4^ Birmingham Business school, University of Birmingham, UK ^5^ Water Aid, UK, ^6^ International Center for Diarrhoeal Disease Research, Bangladesh ^7^ Ministry of Health, Gambia, ^8^ School of Social Policy, Health Services Management Centre, University of Birmingham, UK ^9^ School of Sport, Exercise and Health Sciences, Loughborough University, South Africa ^10^ Developmental Pathways for Health Research Unit (DPHRU), School of Clinical Medicine, University of the Witwatersrand, South Africa ^11^ Faculty of Infectious and Tropical Diseases, London School of Hygiene and Tropical Medicine, UK
**Name and contact information for the trial sponsor**	Université des Sciences, des Techniques et des Technologies de Bamako. Hamdalaye ACI 2000 Rue:405 Porte:359 BP:E 423 Bamako, Mali
**Role of sponsor**	The sponsor has general oversight in the trial management and carries the medico-legal responsibility associated with its conduct

## Introduction

### Background and rationale

Approximately 525,000 children under five die each year from diarrhoeal disease [1]. It remains the fifth leading cause of death among the under-fives worldwide [2], with sub-Saharan Africa accounting for 40% of cases [1]. Besides mortality, diarrhoeal disease also contributes to infant malnutrition, stunting, and developmental delays [3, 4]. One particularly vulnerable group are children starting to eat semi-solid and solid foods (known as complementary foods) [5].

While many diarrhoea-causing pathogens are destroyed during high-heat cooking, the handling and storage of food can still contribute largely through food contamination [6]. The World Health Organization (WHO) advocates for targeted interventions to improve complementary-food safety and hygiene, particularly through the ‘5 key steps’ of food safety, and Hazard Analysis Critical Control Points (HACCP) to identify specific ameliorating behaviours [7, 8]. Interventions to improve complementary-food safety, reduce geophagy, and improve nutritional intake are potentially of great importance for better diarrhoea control and hence growth and developmental outcomes [2, 9]. Interventions targeting these areas together could potentially bring synergy, thus enhancing behavioural reinforcement that could reduce diarrhoea and improve nutritional status of children.

Smaller-scale randomised controlled trials (RCTs) in West Africa and South East Asia exploring the safety of complementary foods have shown that behaviour change interventions which promote complementary-food safety and hygiene can have a positive impact on child health [10–12]. However, advice and education have a limited impact on behaviour change unless accompanied by means to motivate and empower mothers in the community. Such empowerment includes community support and encouragement and a change of social norms [13, 14]. Drama and traditional arts are known to captivate and involve communities, impart sensitive knowledge, and set trends in behaviour [15]. Yet previous interventions targeting diet or diarrhoea have seldom reported to draw on cultural dramatic arts, community assets, and social norms to motivate behaviour change. African communities have a particularly strong cultural heritage to underpin such potential impact. In this project, we will evaluate an intervention that seeks to change behaviours through personal motivation and social support and to empower mothers through improved social status. The intervention aims to enhance motivators for behaviour change of mothers and modify social norms through the use of cultural performing arts as well as community-level campaigns with community events, public pledging, competitions, peer education and support, and engaging community leaders.

This project builds on past work examining behaviour change interventions for complementary-food hygiene in rural areas [12, 16, 17]. We will also incorporate urban areas in this study, which face different challenges with regard to child health and diarrhoea to rural areas, including population density, particularly in informal settlements, and differences in the role of women, families, and hierarchies [18, 19]. In order to extend the scope of the intervention, we have added a nutrition behaviour change component that fits naturally with the complementary-food safety and hygiene intervention at the time when the child is introduced to complementary feeding. The causal pathways through which our intervention components are hypothesised to improve clinical outcomes are represented in Fig. 2. For example, diarrhoea is often a self-limiting condition but can lead to hospitalisation and enteropathy that affects growth. Growth can be improved through a nutrition behaviour change and reduced diarrhoea, while child development can improve through the mediation of both improved nutrition and reduced illness such as diarrhoea.

### Objectives

The aim of the study is to evaluate the implementation and effectiveness of the MaaCiwara complementary-food safety and hygiene and nutrition intervention. The evaluation comprises several objectives:

1. To evaluate the effectiveness of the intervention to promote improved drinking water and complementary-food safety and hygiene behaviour to reduce water and food contamination, and observed diarrhoea
2. To conduct a subgroup analysis to evaluate whether the effectiveness of the intervention on the primary outcomes varies between:
  urban and rural settings, over time between 4- and 15-month post-intervention follow-up.
3. To explore the causal processes that affect the effectiveness, or lack thereof, of the intervention on key clinical endpoints, as mediated by outcomes, such as geophagy, and maternal knowledge and behaviour
4. To evaluate the effect of the intervention on a number of secondary outcomes, including nutrition, geography, maternal autonomy, infections, growth, and development, and explore variations due between urban and rural settings
5. To estimate the cost-effectiveness of the intervention as incremental cost per case of diarrhoea averted and per unit improvements in growth and development

## Methods

### Trial design

We will conduct a mixed-methods, parallel group, two-arm, superiority cluster randomised controlled trial with baseline measures. Clusters (*N* = 120) will be small urban and rural communities, recruited in equal numbers, randomly allocated in a 1:1 ratio to either treatment or control arms stratified by urban/rural status. Regarding the start of the trial timeline as the beginning of data collection, the data collection and intervention will be as follows: Baseline observations will be taken over 3 months (months 1–3). The main part of the intervention will be rolled out over 3–4 months in all clusters and intensely over 35 days in each cluster (months 9–12). A mid-line and an end-line set of observations will be carried out over 3 months each (months 14–16 and 25–27). The mid-line observations will be referred to as 4 months post-intervention and the end-line observations will be referred to as 15 months post-intervention. Sampling within cluster periods will be cross-sectional (the possibility for an overlap has been allowed for) (see Supplementary file 1 for the SPIRIT checklist).

### Participants

#### Setting

Urban clusters will be recruited from Bamako city, the capital of Mali, while rural clusters will be recruited from villages in the surrounding Bamako, Sikaso and Sego regions.

#### Eligibility criteria and cluster design

##### Urban clusters

**Fig. 1:**
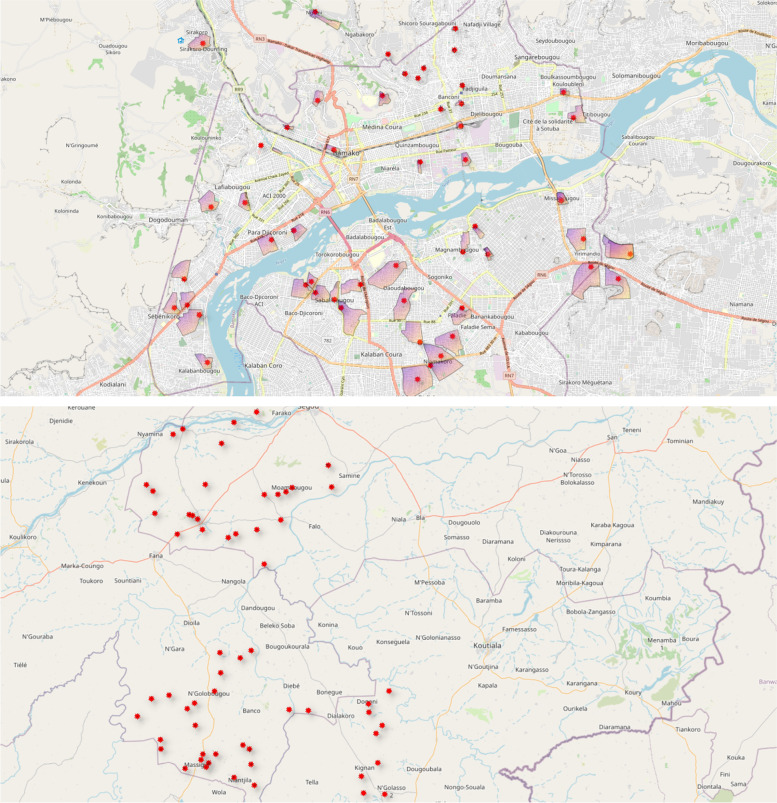
Cluster boundaries and locations in urban Bamako city (top) and rural Bamako region (bottom). Scales differ: villages in the rural region are a minimum of 5 km apart

We created 60 urban clusters in Bamako city (Fig. 1). The clusters are designed to include predominantly poor communities to be approximately comparable to the rural clusters. The clusters are areas of 500 to 2000 people (approximately 200 to 500 households). These clusters are bounded by obvious environmental features including roads and rivers. The clusters are also designed to have a relatively central community area. As much space as possible between cluster areas was included to reduce the risk of contamination, either from individuals in one cluster visiting the intervention in another site or from knowledge produced by the intervention being communicated over larger areas.

##### Rural clusters

Sixty villages were sampled for inclusion from the population of eligible villages (population of 500 to 2000) in Bamako region using a weighted stratified random sample. A sampling frame of all possible villages in each district was constructed. The stratification variables were village size (< = median, > median) and availability of community-led total sanitation. We determined the proportion of villages with and without each characteristic (to act as a weighting factor). Including two stratification variables, each with two levels will result in four strata. The number to be sampled from each group was determined using the weighting factors. Villages were then randomly ordered within strata and approached for inclusion following this random order. Included villages are a minimum of 5 km apart. The location of villages is shown in Fig. 1.

##### Individual participants

Mother–child pairs will be the unit of observation in the study. Within each cluster period, we will aim to recruit 27 consenting mothers with at least one child aged 6 to 36 months (see the ‘Implementation’ section in the ‘Randomisation and sampling’ section).

### Intervention

The intervention will be theory-based upon (Evo-Eco theory and social norms) [20] and will be an adapted version of a community-level complementary-food hygiene and safety intervention previously evaluated in the Gambia, incrementally developed after a series of effectiveness and feasibility trials in Mali, Bangladesh, and Nepal [10–12, 16, 17]. The intervention will also include hygienic play and dietary diversity and meal frequency promotion, which were not included in latter studies, including the Gambia intervention [11, 12, 16, 17]

The intervention schedule and tools used in the MaaChampion Gambian study [16, 17] will be contextualised and adapted by applying the findings of a mixed-methods formative research study conducted in Mali in 2021 in seven rural and urban communities (not included in the trial) and developing a creative brief for a Mali creative team to design the community-level intervention components [11]. The full intervention, once adapted, will be described in a separate publication in compliance with the template for intervention description and replication (TIDieR) checklist and guide [21]. The basic components and principles of the intervention will be the same as the MaaChampion intervention [16, 17]. The adaptation will mainly influence the choice of behaviours, language and cultural aspects of dramatic arts, and the focus on community hierarchies.

Table 1 presents the provisional timeline and steps of the intervention delivery. The intervention will involve 5 days of campaign community visits dispersed across 35 days (days 1, 2, 15, 17, and 35), including home visits, community events, neighbourhood meetings, dramatic arts (songs, drama, stories, animation), and competitions based around achieving a model-mother status. Community volunteer home visits followed between each campaign day, and a reminder campaign day after 9 months to embed the behaviours into social norms, and thereby enhancing sustainability. They will be paid minimally (comparable to local health system community volunteer) for the first year of the project.

**Table 1:** Example timetable of the intervention delivery (T = days from the start of intthe ervention period)

Visits	T	Activity or event
Visit 1	1	Meeting with the community leader
		Community announcement to invite community members to the upcoming afternoon event
		House-to-house visit with community mother supervisors
		Record a short video with a community leader
		Afternoon community event (includes dancing and song, animation, street theatre, public pledging, etc.)
		Training assistants of the “Mother Supervisors”
Visit 2	2	Meeting with the community leader
		Community announcement to invite community members to the upcoming afternoon event
		House-to-house visit with community mother supervisors
		Neighbourhood meeting with demonstrations and stories
No visit	3–14	“Mother Supervisors” and assistants carry out house-to-house visits to encourage and assess mothers
Visit 3	~ 13–17	Meeting with the community leader
		Community announcement to invite community members to the upcoming afternoon event
		House-to-house visit with the community “Mother Supervisors”
		Afternoon community event
No visit	15–24	“Mother Supervisors” and assistants carry out house-to-house visits to encourage and assess mothers
Visit 4	~ 25–32	Meeting with the community leader
		Community announcement to invite community members to the upcoming afternoon event
		House-to-house visit with the community “Mother Supervisors”
		Afternoon community event
No visit	28–40	“Mother Supervisors” and assistants or MaaCiwaras carry out house-to-house visits to encourage and assess mothers
Visit 5	~ 40–45	Meeting with the community leader
		Community announcement to invite community members to the upcoming afternoon event
		House-to-house visit with the community “Mother Supervisors”
		Afternoon community event and certification of village and mothers
No visit	40–270	“Mother Supervisors” and assistants or MaaCiwaras carry out house-to-house visits to encourage and assess mothers
Visit 6 (9 months)	~ 9 m	Meeting with the community leader
		Community announcement to invite community members to the upcoming afternoon event
		House-to-house visit with the community “Mother Supervisors”
		Afternoon community event

Implementation will be through intervention teams who will include district-level community health promotion or public health staff, community traditional communicators (traditional singers, drummers, and performing artists), and respected community members as assisted volunteers (often older mothers or traditional birth attendants). All community members (including fathers) will be involved as well as community leaders in promoting the intervention.

#### Control condition

The control communities will receive a 1-day community-based campaign on the use of water in homes and outdoors. The content is designed to be similar to the intervention in terms of being a community-based intervention delivered through a 1-day campaign event, but not contain equivalent content on food and water preparation, hygiene, child nutrition, or hygienic play. It is designed to represent standard activities. The specific choice of topic for control communities will be finalised after the adaptation of the intervention to ensure minimal overlap. As with the intervention, a Public Health/Health Promotion Officer will provide the visit in each control community. The intervention and control village activities will be delivered in parallel.

### Randomisation and sampling

#### Sequence generation, allocation concealment, and cluster allocation

An independent statistician will generate an allocation sequence using the random generator in Stata v17.0 with assignments to the intervention or control group stratified by rural and urban status. Random block sizes of two and four will be used to maintain both balance within stratum and prevent lack of allocation concealment. Allocation is planned to take place immediately prior to intervention, following baseline data collection in all participating clusters.

#### Implementation

##### Individual sampling

We will recruit 27 mother–child pairs (child aged between 6 and 36 months) per cluster period. Prior to each round of data collection, local community female informants (usually volunteers working with the local health system) will be asked to compile a numbered list of all eligible mother–child pairs in the area (or approximate area in the case of urban) of the cluster. A random ordering of pairs will be generated by the trial statistician in advance of data collection and provided to the field supervisor. The field team will arrive at the cluster the day prior to planned data collection, where they will sequentially attempt to identify each mother–child pair on the list in order until a total of 27 pairs have agreed to participate.

Participating households will be assigned a predetermined unique identifier based on the cluster period and list position. Mother–child pairs who are either not locatable, who reside outside the cluster boundary, or who are not available the following day will be excluded. In post-intervention periods, it is possible some mothers who were in previous rounds may be included. Despite this, we will treat sampling as cross-sectional for three main reasons. There are logistical challenges to tracking consistent trial identifiers across trial data collection periods in this setting. Excluding mothers who have been included in prior survey rounds may significantly limit the available sample size. And statistical analyses incorporating an individual-level random effect to capture cohort effects will not likely be possible as the majority of participants will only be observed once during the study.

##### Informed consent

Initially at the time of cluster recruitment, written and oral permission was sought from the community leader(s) within each cluster. We provided a verbal presentation of the intervention and study to the community leader(s) along with an information sheet on the study. If the community leader could not read, it was read to them by the fieldworker. All materials were translated into French and Bambara and presented in the language of the community leader(s). Community leaders are allowed to withdraw their communities from the study at any time.

Subject to the permission of community leaders, we will approach eligible mothers to participate in the study at each time point. The day prior to any observation or data collection, prospective study participants will be approached and provided with information about the data collection in French or Bambara as required in writing or to be read/explained to them, and consent will be sought (see Supplementary file 2). Mothers are allowed to withdraw from the study at any time.

### Blinding

Due to the nature of the intervention, blinding of mothers and the intervention team will not be possible. However, the mothers will not be informed individually that there is a trial taking place since the consent was taken from community leaders in the Spring of 2021. During the assessment rounds, the data collection will not be linked to the intervention or the trial. The mothers and data collectors will be masked to the nature of the assessment: At baseline and 4- and 15-month assessments, mothers will be informed that the assessment is investigating how children aged 6 to 36 months and their mothers spend their days in rural and urban Mali and to support the delivery of local health and social services. Complementary-food safety and hygiene components of the assessment tools will be concealed in a larger assessment, with a package of observation tools and questionnaires about household food and water use, mother and child activities (including observation and a questionnaire on child care and play activities), health-seeking, water and sanitation, income in the household, and village activities, including sub-study on the use of plastics. Thus, the data collectors will be trained for, and mothers consented to, the conduct of this larger assessment of the household’s food and water consumption, health, and childcare.

Independent field teams will be recruited in each round and will not be informed of the intervention or the inter-village comparison. The statisticians completing the analysis will be also blinded as to treatment allocation.

### Outcomes

**Fig. 2:**
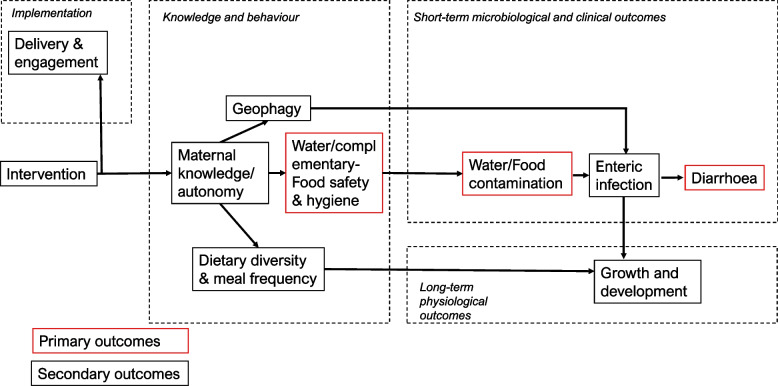
Causal diagram represented as a directed acyclic graph representing the causal assumptions of the study. The three primary outcomes in red are intended to capture the general pathway behaviour → exposure → incident case

The intervention is complex in nature and is designed to target multiple causal pathways that could impact on several child health outcomes. Figure 2 shows a causal diagram indicating the assumed relationships between the different types of outcome. For our main analysis, we focus on a ‘primary causal pathway’ for complementary-food safety and hygiene linking the intervention to diarrhoeal disease via water and food contamination. From this pathway, we include three primary outcomes, one behavioural, one microbiological, and one clinical (to capture behaviour → exposure → incident case) (Table 2).

**Table 2:** List of study outcomes. For each of the outcomes, the difference between the intervention and control groups at 4 months post-intervention and 15 months post-intervention (primary assessment time) will be calculated

Outcome category	Description	Method	Units
**Primary outcomes**			
Water and food safety and hygiene behaviour	The number of events during child’s water and food preparation and handling where the mother has an opportunity to practice a pre-specified drinking water and complementary-food safety and hygiene behaviour in which the behaviour is observed (e.g. hand washing before food preparation) of all total opportunities in the observation period	Observation (number not fixed)	Number of opportunities met (binomial)
Food and water contamination	*E. coli* count in child’s food and water samples	Sample collection and field testing	Colony-forming units/gramme (cfu/g) (count)
Diarrhoea	Observation of stool consistency (watery diarrhoea) [22] by field data collectors and history of at least 2 other such stools in the last 24 h	Observation of collected stool by a fieldworker	Dichotomous
**Secondary outcomes**			
***Implementation***			
Fidelity	Number of visits of the intervention delivered as planned	Investigator report	Count
Uptake	Number of mothers who reported the intervention (Table 1)	Survey	Count
***Knowledge and behaviour***			
Nutrition	Minimum acceptable diet based on minimum dietary diversity and minimum meal frequency they are fed during the day (DHS survey, see below)	Survey	Dichotomous
Geophagy	Number of behaviours observed from total opportunities in the assessment of child play environment and behaviour over the observation period	Observation (number not fixed)	Number of opportunities met (binomial)
Maternal autonomy	Women’ s Autonomy Measure (from DHS survey). Proportion (of *n* = 9) of household decisions the woman participates in	Survey	Number of opportunities met (binomial)
***Short-term microbiological and clinical outcomes***			
Acute respiratory infection	Parental report of cough and difficulty breathing in past 7 days	Survey	Dichotomous
Diarrhoea hospitalisation	In-patient hospitalisation (if given a bed to stay for observation, tests, or treatment for > 3 h) for diarrhoeal disease in the past 3 months	Survey	Dichotomous
Enteric infection	Qualitative PCR of the following pathogens: ETEC, EAEC, EPEC, astrovirus, sapovirus, rotavirus, adenovirus, *Giardia*, *Cryptosporidium*, *Entamoeba histolytica*	Sample collection and lab testing	Dichotomous (for each pathogen)
**Long-term physiological outcomes**			
Physical growth	Weight	On site measurement	Weight for age (*z*-score [WHO International Growth Tables])
Physical growth	Height	On site measurement	Height for age (*z*-score [WHO International Growth Tables])
Cognitive development	ASQ3 score for age	Survey/observation	Count

A causal effect of the intervention on parental water and food safety and hygiene behaviour is a necessary (although not sufficient) condition for the intervention to have a causal effect on food and water contamination. Our interpretation of results in this study is therefore conditional, both on the explicit causal model and assumptions, and that the interpretation of effects of the intervention on more ‘downstream’ outcomes is conditional on effects observed on mediating outcomes. We also note that diarrhoea can be an unreliable and often very noisy outcome for trials of this nature [23], so the inclusion of outcomes on which it is causally dependent will aid to minimise the risk of faulty interpretation.

We recognise that in a larger community there would be a ‘virtuous circle’ of lower infection rates and reduced transmission. The causal diagram therefore represents an individual-level and relatively simplified set of causal assumptions.

The remaining outcomes are considered ‘secondary’ for the main analysis of the trial, although we will perform a full structural model analysis as an additional analysis to compare to the main analysis to facilitate the causal interpretation of the results. Within each category of outcome, we will also collect several alternative measures, although only one measure from each outcome type will be used for the main analyses described here (as listed in Table 2). A full table of all the primary and secondary outcomes is provided in Table 2 and the alternative measures we will collect are detailed in the Supplementary file 3. We also note that the knowledge and behaviour outcomes can be conceived of as ‘process outcomes’ in alternative terminology. The trial can therefore be conceived as a hybrid implementation-effectiveness study of a complex intervention [24].

#### Data collection procedures

##### Field data collection, food, water, and stool samples

**Fig. 3:**
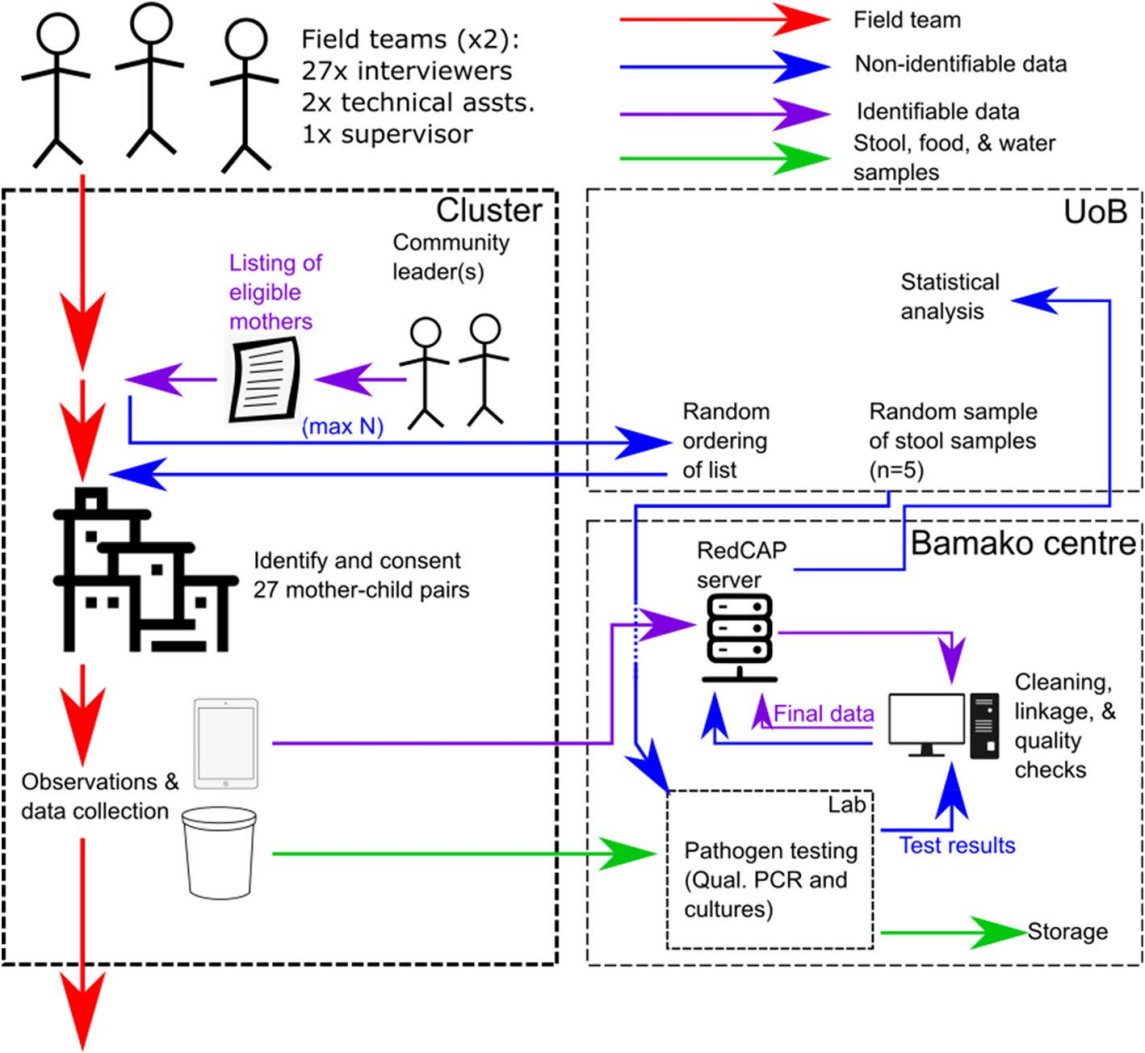
Illustrative flow diagram showing data collection procedure and data and sample flows

Figure 3 shows an illustration of the general data collection process for each cluster per period. Field teams will comprise 27 data collectors (one per mother–child pair), three technical assistants to do the anthropometrics and some laboratory work, and two field supervisors.

The day following the enrolment of participants (see the ‘Implementation’ section), a data collector will spend an average of 8 h with each participating mother and child to capture the various direct observations and complete the survey questions listed in Table 2. Data capture will be on a tablet device using RedCAP software; the server aggregating responses will be hosted at USTTB, Bamako. The field supervisors will conduct spot checks and sit-ins with each data collector throughout the day to check for the quality and accuracy of recorded responses.

In every household, each data collector will provide the materials (cotton underwear/liner, nappies, or potty) to the household. When the child defaecates, regardless if the child has diarrhoea or not, during the data collector’s 7–9-h observation visit, a sample will be collected using aseptic techniques.

We will also collect six water and six food samples from each cluster per period. The samples will be collected from the complementary food and water provided to the child just before consumption from the vessel or spoon or hand of the carer before it goes in the child’s mouth by the data collector using aseptic techniques. Samples can be collected from either breakfast or lunch meals. The field team will be provided in advance with a random sample of unique household identifiers for each cluster period that indicate which households and meal a sample should be taken from.

Stool samples will then be stored in liquid nitrogen capsules and water and food samples in cool boxes for transportation to the Bamako laboratory on a daily basis for analysis following the completion of the data collection in the cluster. Each sampling container will have a unique predetermined anonymous ID and barcode, which will be recorded in the main data collection form.

#### Laboratory procedures

The principal outcome at this level is the prevalence of infection of ten common pathogens in stool and *Escherichia coli* for food and water as an indicator for faecal contamination.

##### Food analysis

Ten grammes of the sample will be weighed and suspended in a sterile vial containing 90 ml of sterile Maximum Recovery Diluents (1:10 ratio). The sample will then be vortexed for 5 min to dislodge bacterial cells from the food particles. The homogenised sample will be allowed to stand for 10 min so that heavy food particles could settle before aseptically transferring 1 ml of the food supernatant to the surface of the Brilliance *E*. *coli*/coliform agar. The sample collected will be spread by pressing gently with the spreader. The inoculated plates will be incubated for 24 h ± 1 h at 37 °C ± 1 °C. Furthermore, all the samples with overgrowth due to heavy coliform contaminations will be serially diluted to countable growth.

We will calculate the total number of coliforms per gramme by multiplying purple and pink colonies by the dilution factor. The number of presumptive *Escherichia coli* will be obtained by multiplying the number of purple colonies by the dilution factor. The coliform count will be expressed in cfu/10 g unit.

##### Water analysis

Ten millilitres of each water sample will be filtered using a membrane filter (0.45 μm pore size). The filtered membrane will be transferred onto an absorbent pad soaked in lauryl sulphate. The inoculated Petri dish will be labelled and incubated at 37 °C for 24 h to isolate the coliforms. We will be only counting yellow colonies using the horizontal grid lines. Coliform count will be calculated by coliform colonies multiplied by 10 (Oxfam Delagua user manual).

##### Stool analysis

DNA will be extracted from stool samples using extraction protocols specific to the pathogens being tested for. Extracted DNA will then be mixed with diagnostic primers for the target pathogens, amplified, and run through gel electrophoresis.

The principal outcome at this level is the prevalence of infection: ten common pathogens in stool and *E. coli* for food and water. Only five stool samples will be tested per cluster period with the remainder stored indefinitely for future analyses. A random sample of unique IDs will be provided to the lab in advance of the study.

##### Data processing and final dataset

Data will be downloaded and checked for consistency and errors daily when field activities are ongoing. Results from the lab procedures (see below) will be recorded on a separate form on the tablet, linked to the ID, and submitted to the server. The raw data will be identifiable as it will contain the GPS location of the household where the data collection took place alongside an identifier that is linked to the name of the participant. Only the data manager in Bamako will have access to these identifiable data. At the completion of each round of data collection, a dataset will be created combining the field and lab datasets. Individual identifiers will be anonymised and an identifiable dataset (containing GPS location) and an anonymised dataset will be generated. Access to the identifiable dataset will be limited to those requiring it for analyses approved by the MaaCiwara Management Committee. Both datasets will be made available to other researchers upon reasonable request. At the end of the trial, only the final datasets will be retained.

### Analysis

#### Statistical analysis

We will conduct two types of analysis. Firstly, the main analysis will follow standard recommended practice for cluster randomised trials. This analysis will be completed using an intention-to-treat (ITT) approach. The primary analysis will consist of the estimation of overall treatment effects and the secondary analysis will consist of a subgroup analysis. We will not use statistical significance in either reporting or for interpretation of intervention effect estimates. Our interpretations will be based on the sizes and uncertainty of treatment effect estimates. Moreover, interpretation of an estimated treatment effect is conditional on the estimated effects of the intervention on causally antecedent outcomes. For example, if there are only small effects of the intervention on water and food safety and hygiene behaviour, then any subsequent effects on food and water contamination must also be small. The conditional interpretation of our primary outcomes provides some protection against ‘type I errors’ of interpretation and so we therefore also do not opt to adjust for multiple outcomes [25]. The primary analysis will be unadjusted for covariates except those used in the randomisation (rural or urban status) and a sensitivity analysis will adjust for a pre-specified set of covariates (see covariate-adjusted analysis below).

Additional analysis will take a more explicit structural equation approach to account for the dependencies we assume in the data (Fig. 2) and to address objective 3, which is to estimate the effects of the intervention (or lack thereof) mediated by different potential causal pathways. However, this more complex analysis can fail (e.g. convergence failure) and so it is left as an additional analysis. We describe each analysis in turn.

##### Descriptive analysis

The study population will be summarised at the cluster level and individual level. Characteristics of clusters and individuals will be summarised by intervention and control groups and stratified by time period (baseline and 4 months and 15 months post-intervention). Categorical data will be summarised by counts and percentages. Continuous data will be summarised by means and standard deviations or medians and interquartile ranges, as appropriate.

##### Primary analyses

All outcomes will be summarised by intervention and control groups. We will estimate overall pooled (urban and rural) effects for all three primary outcomes at 4 months and 15 months post-intervention (primary assessment time is 15 months). We will analyse each outcome using a generalised linear mixed model. The level of observation is the individual nested within cluster periods. Models will include an intercept, an indicator for whether the cluster had the intervention at the time, and a post-intervention time period indicator. We will also include the covariate used in the randomisation process in each model. Normally distributed random effects with an unknown variance will be included at the cluster and cluster period levels.

For binary and binomial outcomes, we will use a generalised linear mixed model with a binomial distribution and logit link to estimate odds ratios. For continuous outcomes, we will use a generalised linear mixed model with a Gaussian distribution and identity link to estimate a mean difference. For count outcomes, we will use a generalised linear mixed model with a Poisson distribution and log link to estimate rate ratios. If there is overdispersion in the count outcome, a negative binomial distribution with a log link will be used to estimate rate ratios. For non-linear models, the absolute differences will be averaged over the study population (the average marginal effect) using a marginal standardisation approach. All estimates will be reported with 95% confidence intervals and *p*-values associated with a two-sided test of no difference.

The model assumes a cross-sectional sampling structure within cluster periods. If there are a high proportion of mothers appearing in multiple rounds (with the same or different children), and we can successfully link observations, then we will treat the outcomes as repeated measures and include a mother-level random effect term. We will also report estimated within- and between-cluster variance. For the primary outcomes, the analysis is not adjusted for any covariates so there will be no missing covariate data.

##### Covariate-adjusted analysis

We will conduct the analysis for the primary outcomes adjusting for the following covariates: population size (< / ≥ median), presence of community-led total sanitation in rural areas, and whether there is a school located in the cluster. For this analysis, if there is more than 5% missing data in the covariates, multiple imputation will be performed accounting for clustering. The number of imputations performed will be based on the percentage of missing data in the covariates used.

##### Secondary outcomes

All secondary outcomes will be summarised by intervention and control groups. We will estimate the overall pooled effects for the secondary outcomes at 4 months and 15 months post-intervention using the same statistical approach as for the primary outcomes. Both unadjusted and adjusted models will be fitted. No subgroup analysis will be performed for the secondary outcomes.

##### Subgroup analysis

We will conduct a subgroup analysis to compare the effect of the intervention in urban and rural settings at 4 and 15 months post-intervention (primary assessment time). We will add an interaction between an indicator for urban or rural clusters and the treatment effect. Estimated effect sizes in both relative and absolute terms for urban and rural clusters will be reported along with 95% confidence intervals.

#### Additional analysis

##### Structural model

Our additional analysis will take an exploratory and explicitly structural approach, and we will fit a series of models based on the assumed causal relations specified in Fig. 2 to facilitate the interpretation of the causal effects of the intervention. For this analysis, we will use Bayesian methods, given the complexity of the model structure. The models will account for clustering and an individual-level random effect will also be added if there is a high proportion of women in multiple rounds of observations. The random effects will allow for correlation between the outcomes at an individual and cluster level. We will use weakly informative priors for the model parameters and hyperparameters. Estimated treatment effects as stated in the primary analysis will be reported with 95% credible intervals.

The structural model will enable us to extract several effects of interest. In particular, the effects of the intervention on enteric infection and growth as mediated by the different pathways in the diagram [26]. One comparison of particular interest is whether the effects of the intervention differ in urban and rural areas; thus, this analysis will take place separately for urban and rural clusters.

### Sample size and power

We plan to use stratified randomisation to allocate 120 communities to two arms and recruit 3240 mother–child pairs (27 per cluster period) in total at baseline, mid-line (4 months post-intervention), and end-line (15 months post-intervention) as three cross-sectional data collection rounds. For the three primary outcomes, we will collect:

1. Water and food safety and hygiene behaviour: a binomial outcome with four questions per pair for all 27 mother-child pairs
2. Food and water contamination: a count outcome from 10 samples randomly selected from the 27 total samples (1 per pair) will be analysed
3. Diarrhoea: a single dichotomous observation for each of the 27 mother-child pairs

We report the power for the three primary outcomes listed in Table 3 for a range of effect sizes assuming an intraclass correlation coefficient of 0.02, a cluster autocorrelation coefficient of 0.8, and a type I error rate of 5%. Generation of ICC and CAC estimates follows recommended guidance [27], and we have used values informed by similar outcomes in similar settings for the ICC (noting that ICCs for process outcomes tend to be higher for those for clinical outcomes) [28]. For the CAC where we have limited information on values, we have used the values of 0.8 and 0.9 as recommended in the literature [29]. We consider sensitivity to a range of plausible values: power calculations with alternative values of the ICC and CAC are provided in Supplementary file 3. We used the method described by Hemming et al. [30] to determine study power for the primary outcomes, and for the subgroup analyses, we adapted this method to that reported by Demidenko [31]. We did not include covariate effects nor the randomisation procedure in the power calculations; thus, these results can be considered conservative. Since the outcomes will be evaluated using repeat cross-sectional rounds, loss-to-follow-up of specific individuals will not be an issue. The sample size calculation was performed using an RShiny app for cluster trials [https://clusterrcts.shinyapps.io/rshinyapp/].

**Table 3:** Power for the three main outcomes for different combinations of effect sizes and assuming an ICC of 0.02, a CAC of 0.8, and a type I error rate of 0.05. Power for non-linear models calculated using a normal approximation. *pp* percentage point

**Outcome**	**Assumed model**	**Assumed baseline**	**Effect size**		**Obs. per cluster period**	**Power**
*Primary outcomes*						
Water and food safety and hygiene behaviour	Binomial-logistic	50%	+ 5 pp		27 mother–child pairs 4 opportunities per pair	69%
			+ 10 pp			> 99%
			+ 20 pp			> 99%
Food and water contamination	Poisson	10 cfu/g	− 1		10 samples per cluster	> 99%
			− 2			> 99%
Diarrhoea	Binomial-logistic	13%	− 2 pp		27 mother–child pairs	33%
			− 3 pp			64%
			− 5 pp			97%
			− 7 pp			> 99%
**Outcome**	**Assumed model**	**Assumed baseline**	**Main effect**	**Interaction effect**	**Obs. per cluster period**	**Power**
*Subgroup analyses*						
Water and food safety and hygiene behaviour	Binomial-logistic	50%	0 pp	+ 5 pp	27 mother–child pairs 4 opportunities per pair	67%
			0 pp	+ 10 pp		> 99%
			+ 5 pp	+ 5 pp		69%
			+ 5 pp	+ 10 pp		> 99%
Food and water contamination	Poisson	10 cfu/g	0	− 1	10 samples per cluster	29%
			0	− 2		84%
			− 2	− 1		34%
			− 2	− 2		91%
Diarrhoea	Binomial-logistic	13%	0 pp	− 2 pp	27 mother–child pairs	11%
			0 pp	− 5 pp		54%
			− 2 pp	− 2 pp		13%
			− 2 pp	− 5 pp		65%
			− 5 pp	− 2 pp		18%

#### Qualitative analysis

In parallel to the trial, comparative focussed ethnographic research will be carried out in a random sub-sample of the control (2 × rural, 2 × urban) and intervention (3 × rural and 3 × urban) clusters, with a focus on understanding the social, political, and cultural context in which the intervention is being implemented and the acceptability of the intervention in this context, particularly the local arts-based campaign approach. The qualitative analysis will enable a more nuanced interpretation of issues of potential scalability and transferability to different cultural contexts, through refining the programme’s theory of change. If the intervention is found to be effective, it will be presented to the necessary stakeholders for scaling up in other settings may include the control communities. An external advisory group already engages implementing and funding stakeholders in Mali and some globally.

The primary method will be participant observation, via key informants (local artists and families in the selected clusters). The ethnographic fieldworkers will be independent of the intervention delivery and trial outcome measurement teams and will be clear with participants that they have separate objectives. Field notes will be taken using proformas: handwritten notes will be taken in the field, then typed up to create a full set of field notes within 24 h of leaving the field. Field notes will be cross-checked during debrief with other team members and clarifications and reflections added. Supplementary visual methods (photographs, sociograms) and ethnographic (unstructured) interviews will be used when needed to offer further insights or explanations of observed events. Data from these methods will be incorporated into field notes.

In addition, after completion of the trial, up to 12 focus groups will be conducted with mothers, fathers, grandmothers, and village elders in a sub-sample of intervention clusters (different to the ones selected for the ethnography sub-analysis) to draw out their reflections on the intervention process and impact.

Data analysis will take place alongside data collection guided by a realist theoretical approach, with a focus on understanding the influence of context on the mechanisms of action in the intervention. This may include themes such as (i) the influence of urban and rural context upon intervention adaptation and implementation, (ii) the role of cultural (African) dramatic arts beyond information transfer to a mediator for behaviour change, (iii) mother’s empowerment, (iv) implementation processes, and (v) social network/support mapping.

We will be working across numerous languages (local dialects, French, English), and therefore, we will use the Framework method for the management and analysis of qualitative data. This method is appropriate in this context because it requires the production of summarised data (field notes and/or transcriptions), which can then be translated into French and English and then compared and contrasted more easily between themes and sites.

#### Health economic analysis

There is very little economic evidence about sanitation and hygiene programmes in general [32] or on the cost-effectiveness of particular types of intervention [33]. The primary base case analysis will adopt a societal perspective as far as possible. Resource use data will be collected prospectively to estimate the costs in each of the trial arms [34]. This will include costs incurred by the agencies responsible for setting up and delivering the intervention and for the households in the intervention and control villages. Costs associated with developing and delivering the intervention will be collected via trial reporting mechanisms. Costs to the household associated with episodes of childhood diarrhoea and other illness will be captured via a questionnaire which will include healthcare resource use, impacts on household productivity and other costs. The questionnaire will be delivered to participants in the trial during two follow-up visits. We will use established methods to estimate the costs associated with caring for an ill child [35]. The potential costs for the participants associated with the behaviour change promoted will also be examined.

We will evaluate whether the intervention is cost-effective by extending from the framework proposed by Borghi et al. [36]. For the base case, we will assess cost-effectiveness based on the primary trial outcome and assess the incremental cost per case of diarrhoea avoided at 15 months post-intervention. Where possible, we will aim to collect data to enable a broader analysis of costs and benefits (for other health outcomes and beyond health). A range of sensitivity analyses will be undertaken to explore the impact of uncertainty on the study results [37].

### Ethics

#### Research ethics approval

The study received full ethical approval from the University of Birmingham Science, Technology, Engineering, and Mathematics (STEM) Research Ethics Committee (ERN_20-0625). Also, in Mali, full ethical approval was granted by the Faculty of Medicine, and Stomatology, and Pharmacology, University of Science, Techniques, and Technologies of Bamako (Letter Number 2020/253/CE/FMOS/FAPH).

#### Protocol amendments

Amendments to the protocols can only be made by the chief and/or principal investigators or their designees, with agreement from the sponsors. Substantial amendments will also be reviewed by the project’s oversight committees, stakeholders, and must be approved by the Malian ethics committee. Non-substantial or administrative amendments will be documented but do not require formal approval.

#### Data monitoring committee

We will not specify any ‘stopping rules’ for the trial as the intervention is community-based, non-invasive, and non-clinical and presents no risk of harm to the study participants. The role of the Data Monitoring Committee is therefore to monitor trial conduct and data quality rather than identify safety events. The Trial Steering Committee will also constitute the Data Monitoring Committee for the trial and so will adopt its responsibilities and is comprised of independent experts in the area of WASH interventions and their evaluation. The Steering Committee will meet six times over the 3 years the trial is taking place. Prior to trial commencement, it will advise on the design of the study and analysis. Following the completion of baseline data collection, the committee will review a summary of the data collected and the data quality indicators to be specified. The trial statistician in Bamako will prepare this report and analysis. The Committee will also meet to repeat the review following the completion of each subsequent wave of the study.

#### Community engagement and involvement

To improve the safety and cultural appropriateness of this trial, lay individuals from target populations will be substantially involved in the research, through formative research and throughout the study in the following ways:

1. Community leaders and mothers in communities were and will continue to be consulted during the development of the study, in both rural and urban Mali.
2. Formative research took place through running focus groups and home visits with community members, such as mothers, fathers, grandmothers, and community leaders. The working group that will adapt the intervention will include non-researchers who are themselves from communities: a creative team with local traditional communicators and dramatic artists and public health officers working in the existing Mali community health system. This allows for the adaptation of the intervention and the survey instruments to the community, allowing for the study to be as acceptable and relevant as possible.
3. The trial’s Mali Expert Advisory Group will include two community members to provide ongoing support and advise on the management of research, undertaking of research, and analysis and interpretation of results.
4. An extensive list of stakeholders and policymakers (and community members at the end of the study) will also receive update newsletters and the results of the study through the Communication and Dissemination Plan, encouraging them to feedback and send views and questions. The trial website will also allow for such interactions through different contact routes in Mali and UK.

## Trial status

1.1 07 Dec 2021—The trial protocol was submitted to medRxiv as a preprint https://doi.org/10.1101/2021.12.15.21267512. 

1.2 31 Mar 2022—Amended funding statement to include support from the National Institute for Health Research (NIHR) Applied Research Collaboration (ARC) West Midlands.

Recruitment for baseline evaluation began on 12 January 2022 and will finish in May 2022.

Intervention implementation is to begin in October 2022.

### Supplementary Information

**Additional file 1.** SPIRIT checklist for Trials.

**Additional file 2.** Community Leader Information and Consent Form.

**Additional file 3.** Supplementary tables.

## Data Availability

The custodian of the final dataset will be the PIs. Data will be controlled by selected staff in both Mali and the UK. The Mali and UK teams will maintain exclusive use of the data until the data are published in an open-access journal. Afterwards, data will be made available by formal request to the custodian.

## Abbreviations

STEM: Birmingham Science, Technology, Engineering, and Mathematics
TIDieR: Template for intervention description and replication
RCTs: Randomised controlled trials
HACCP: Hazard Analysis Critical Control Points
